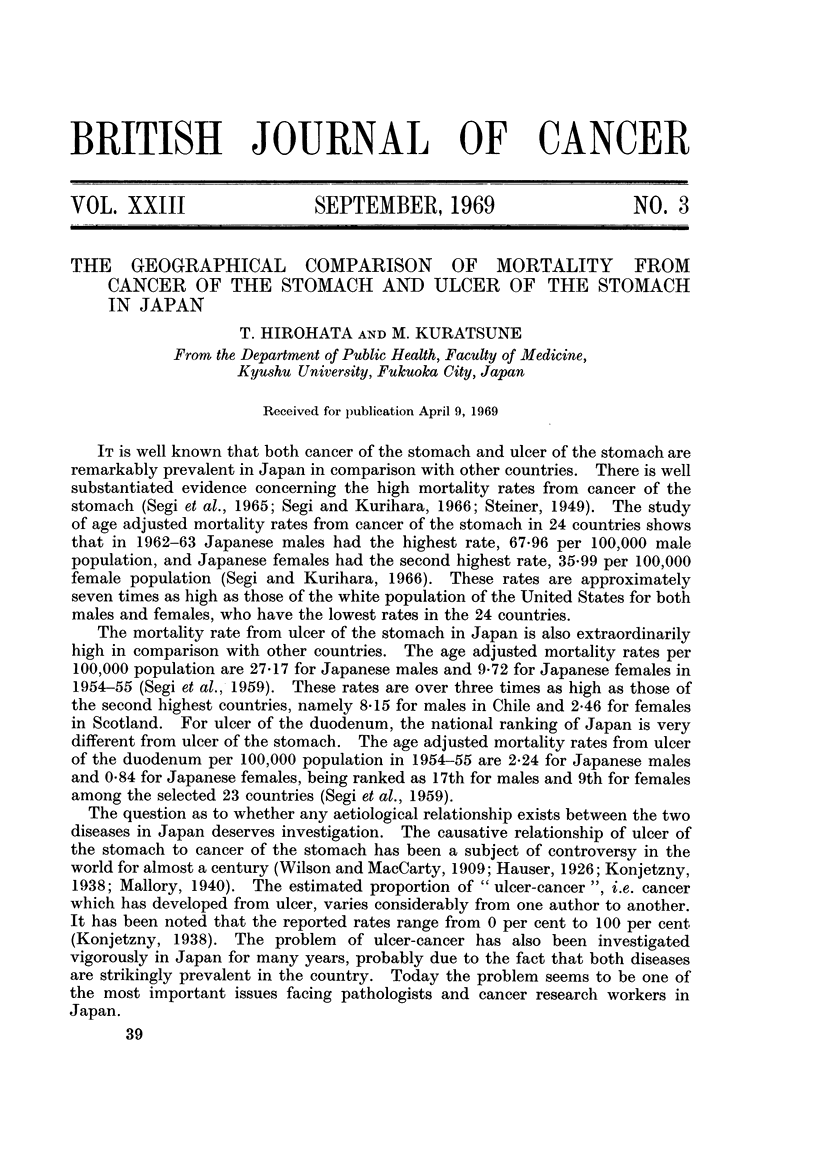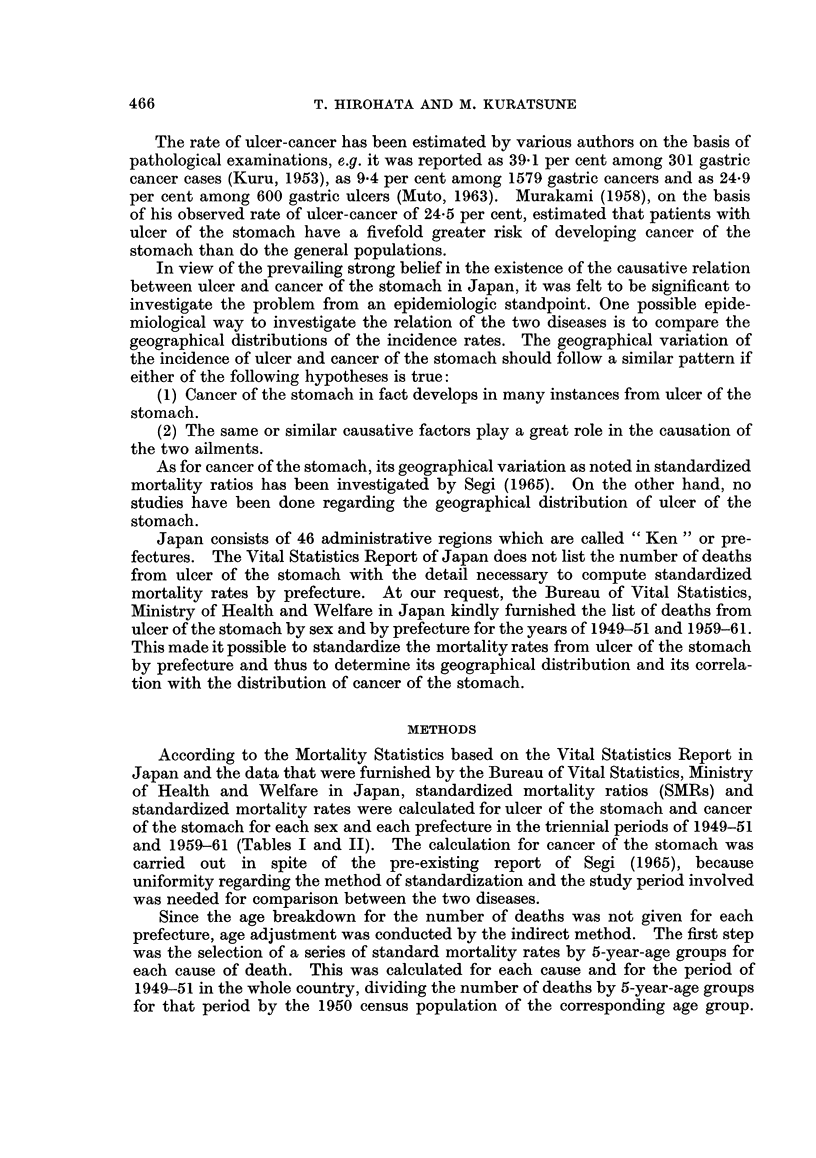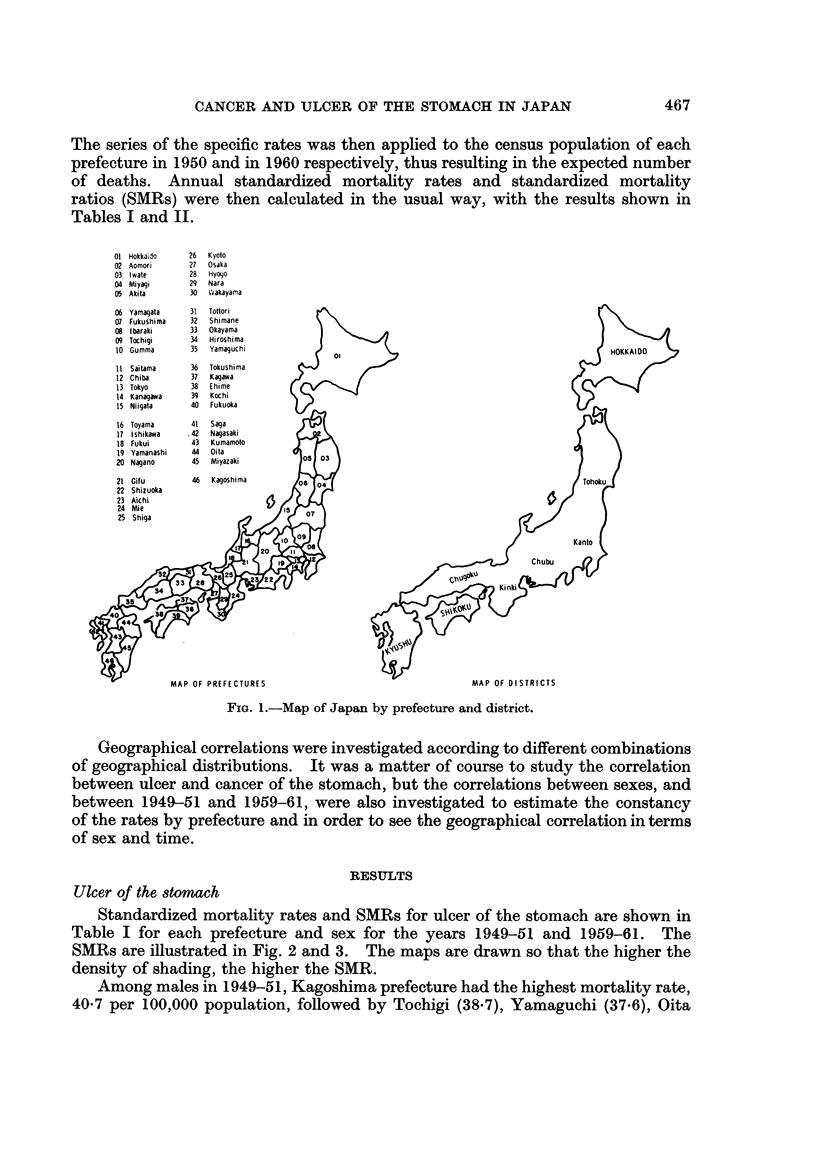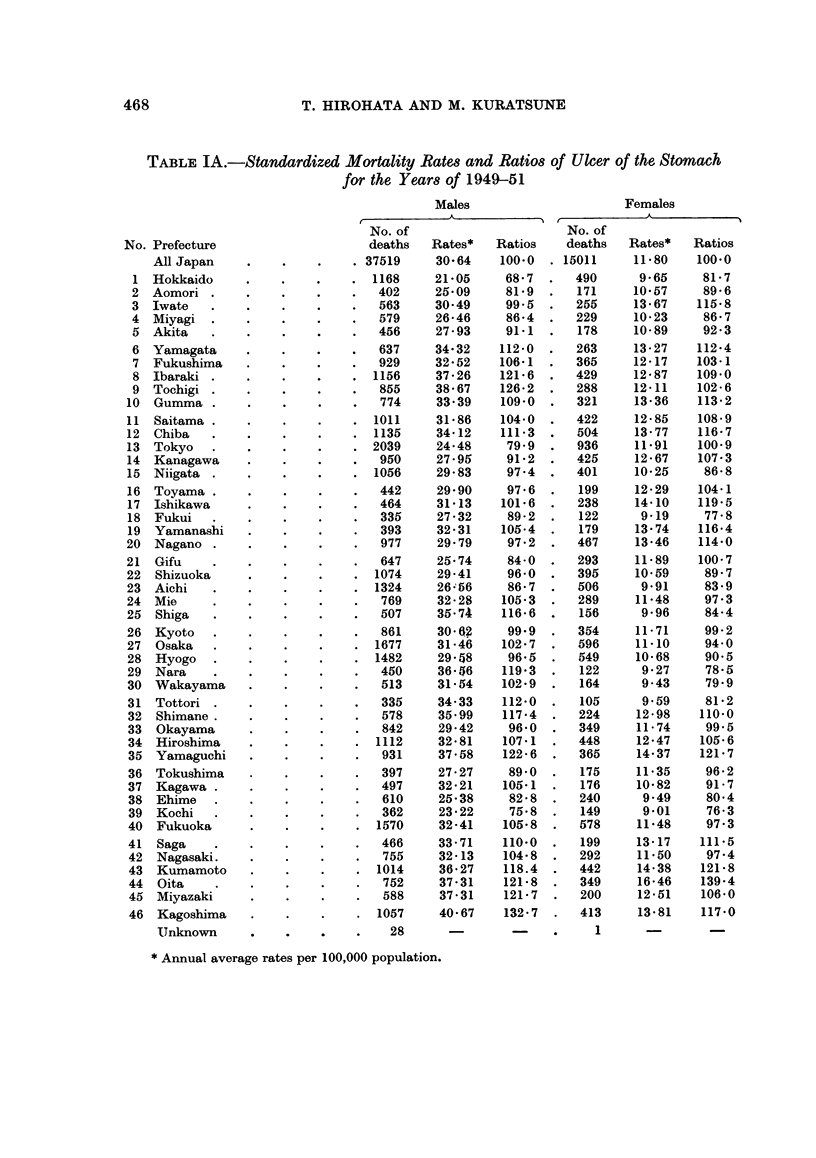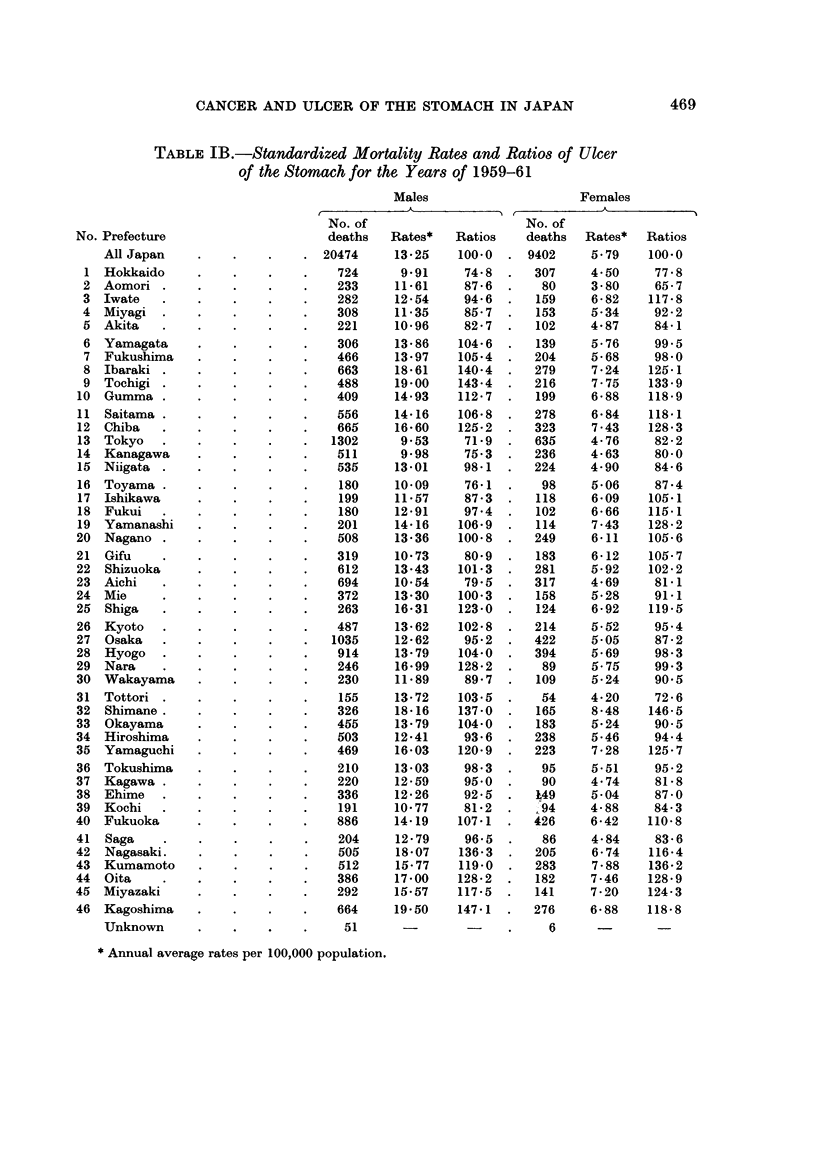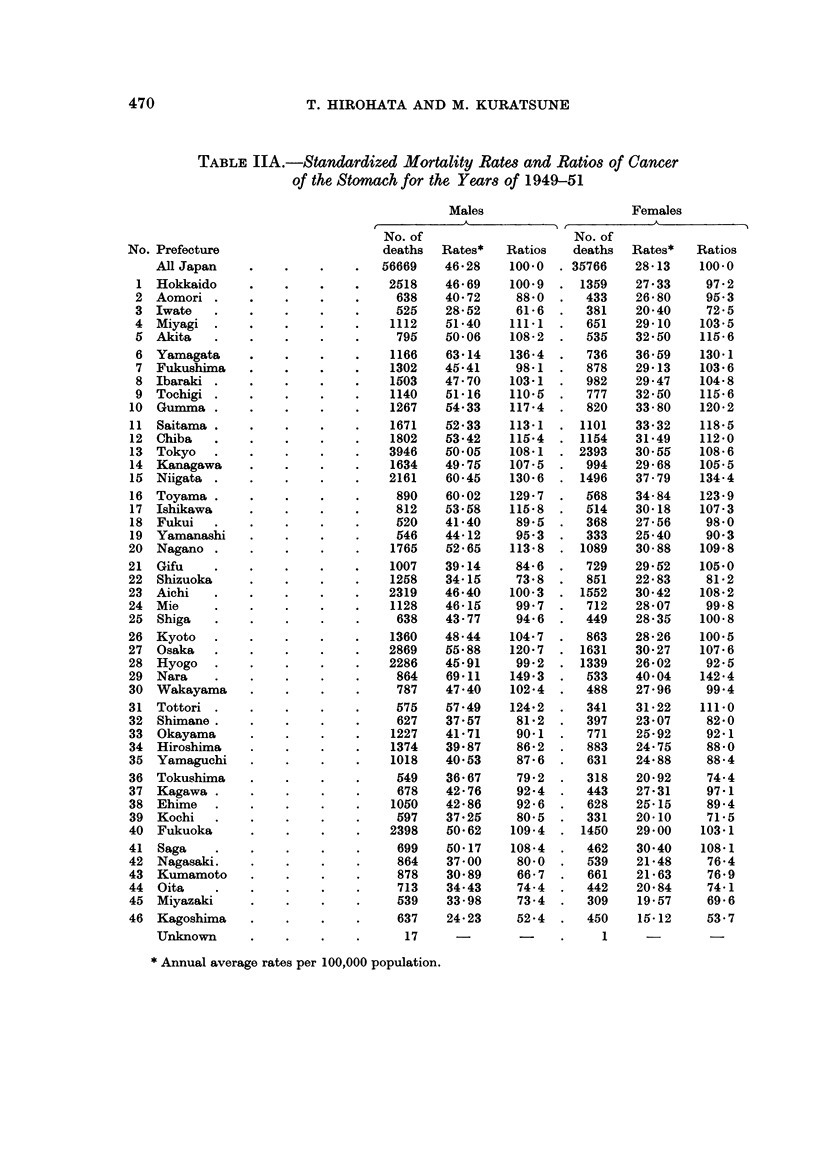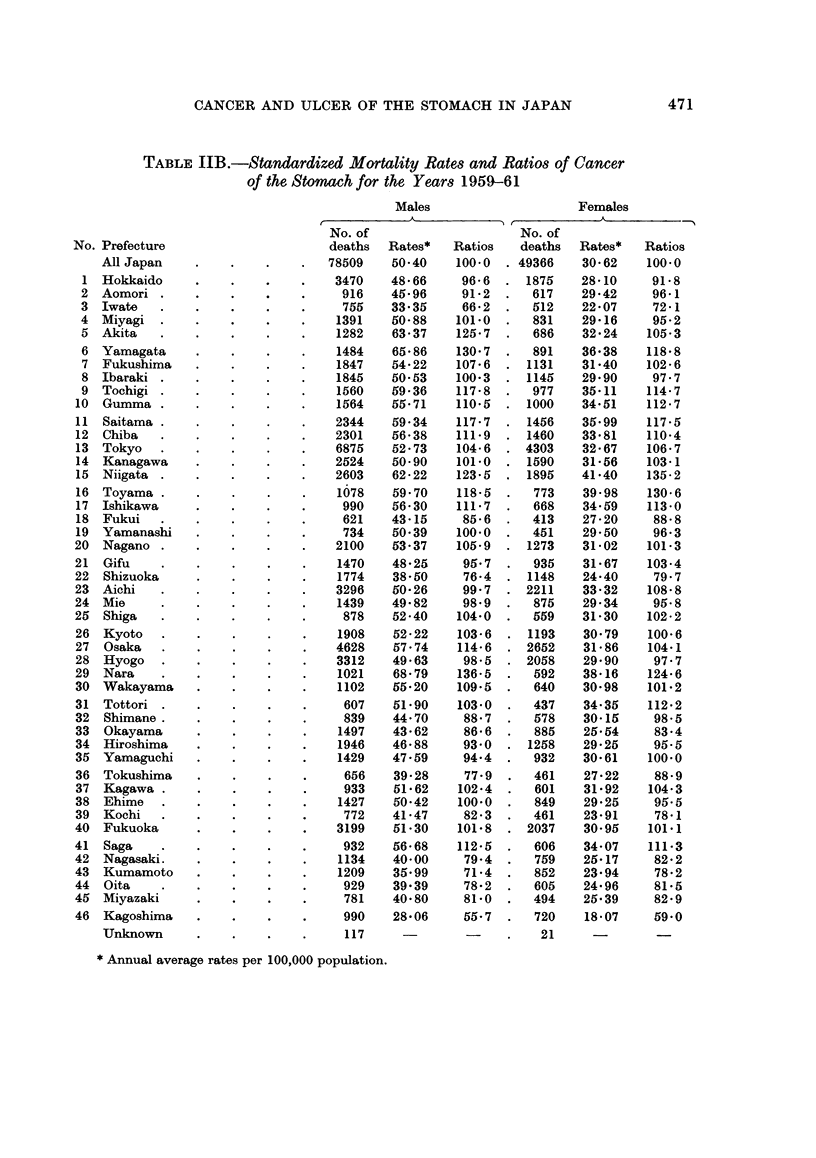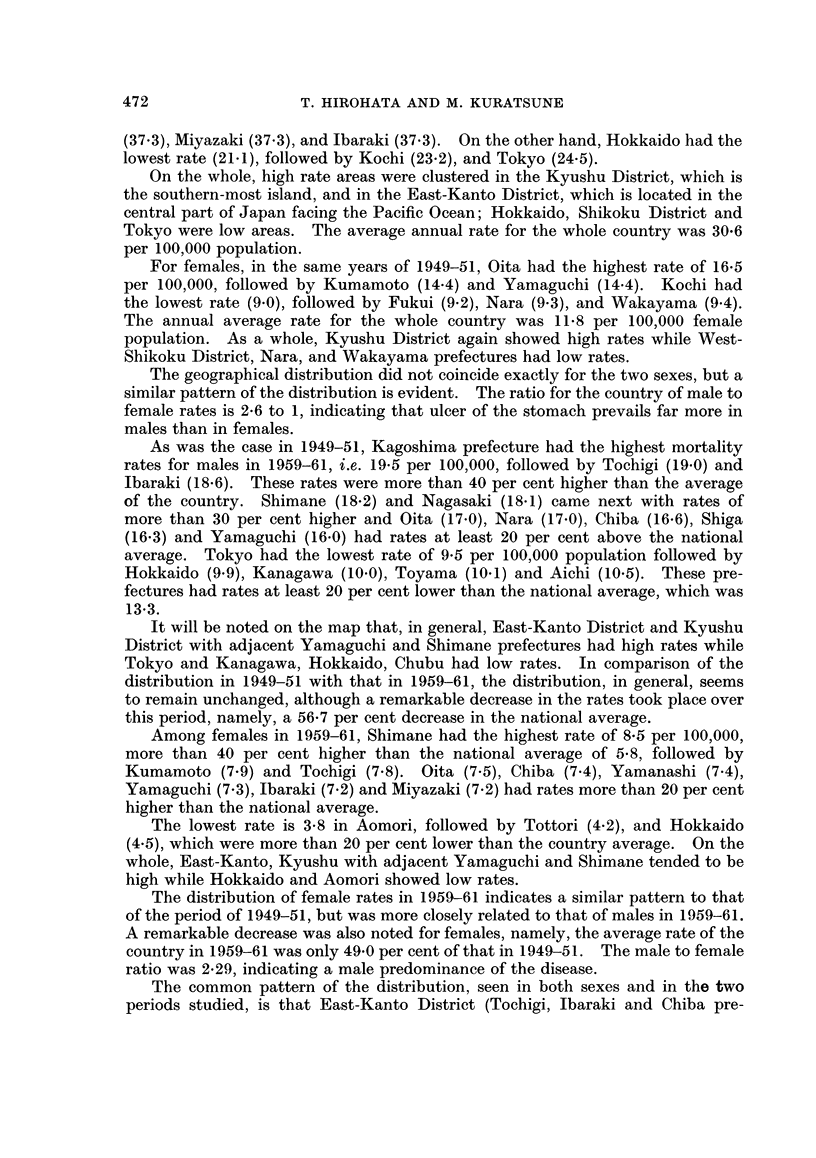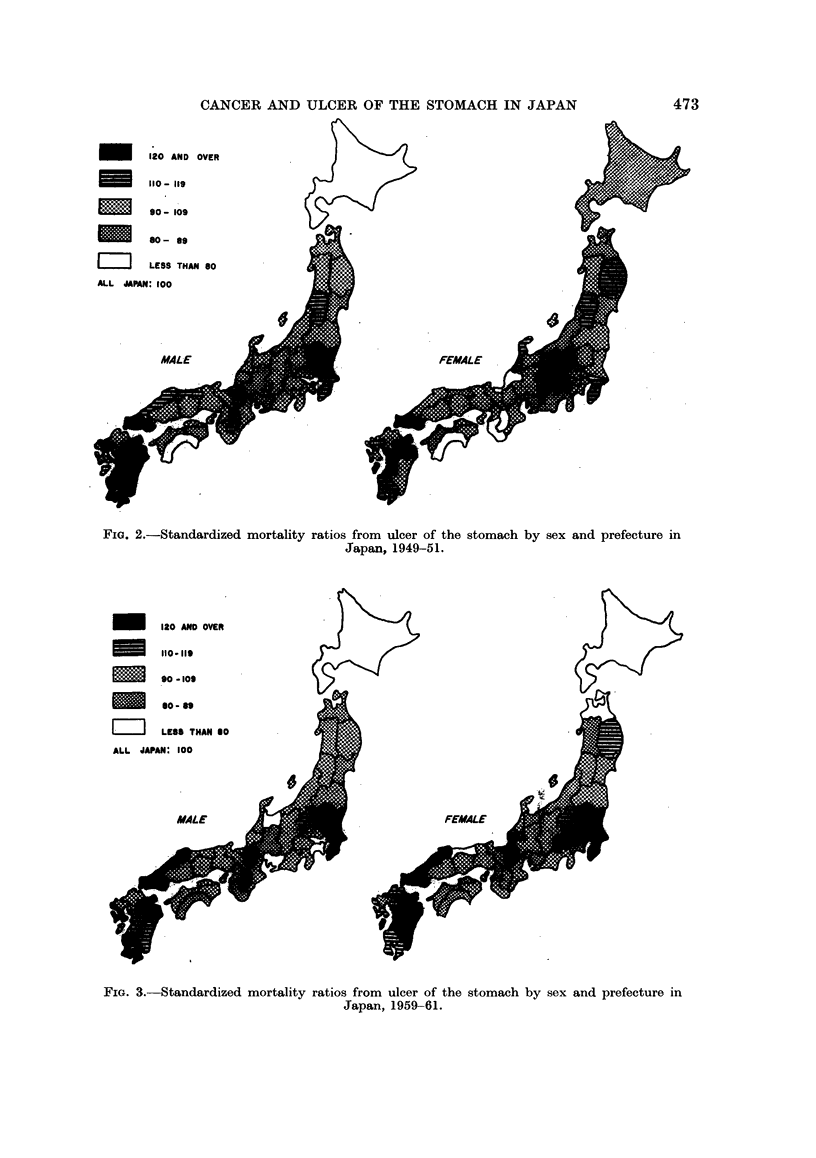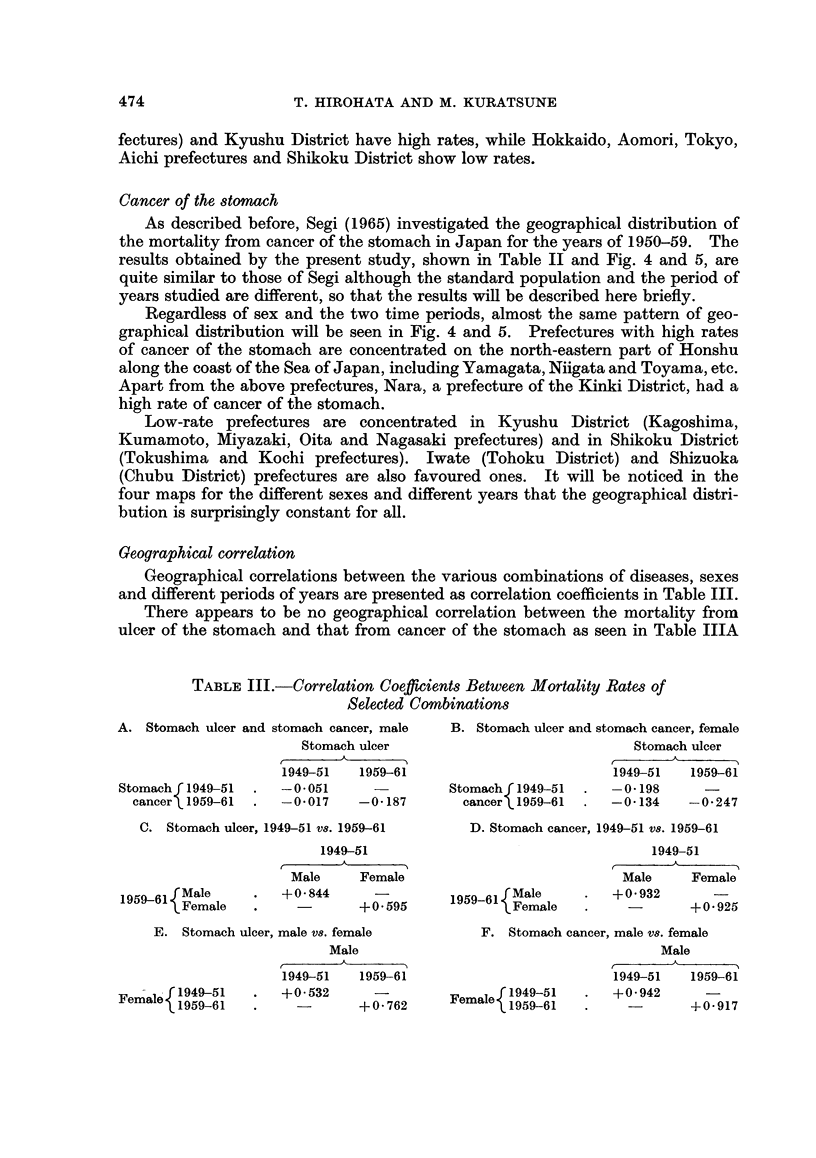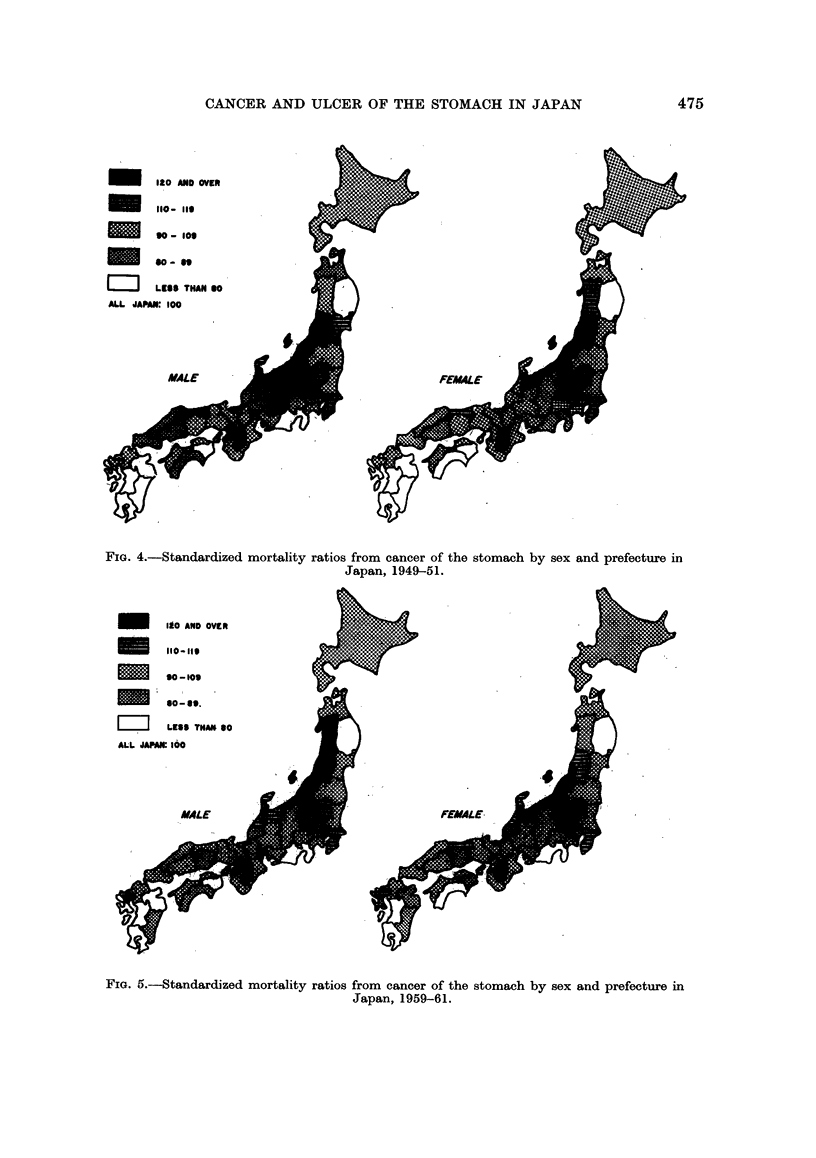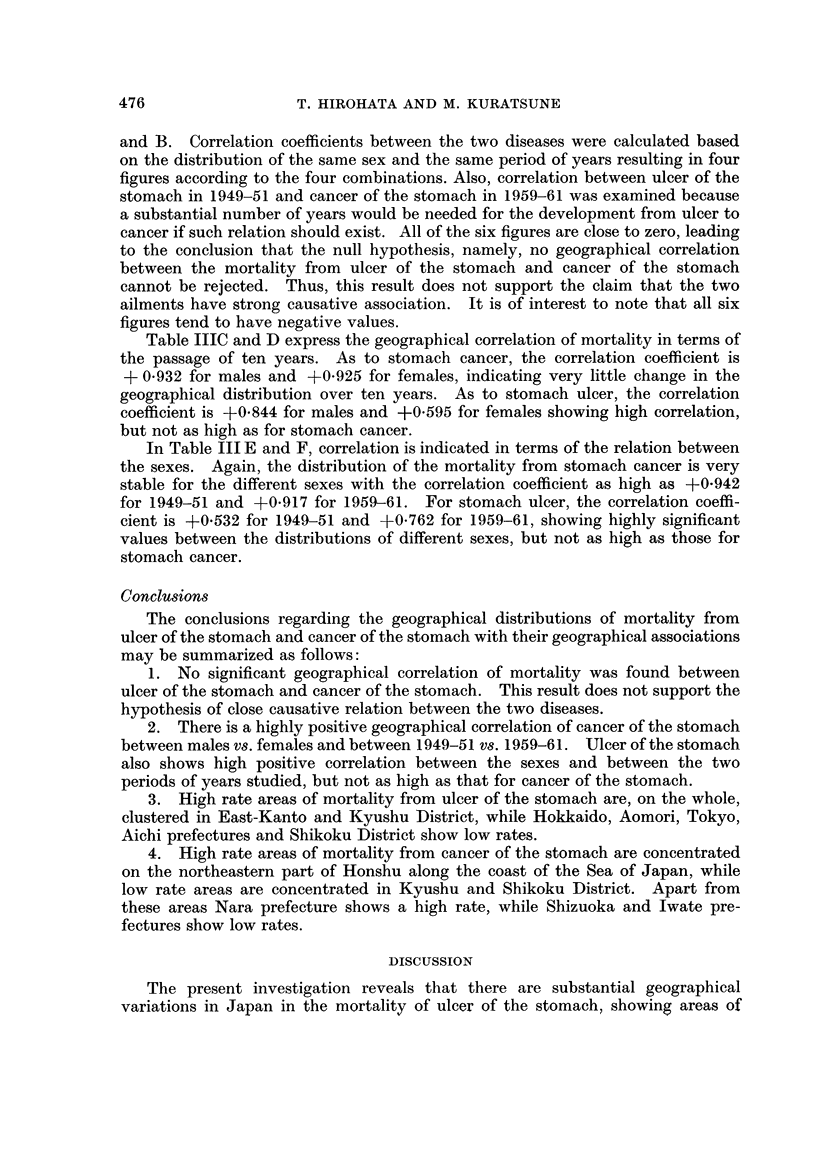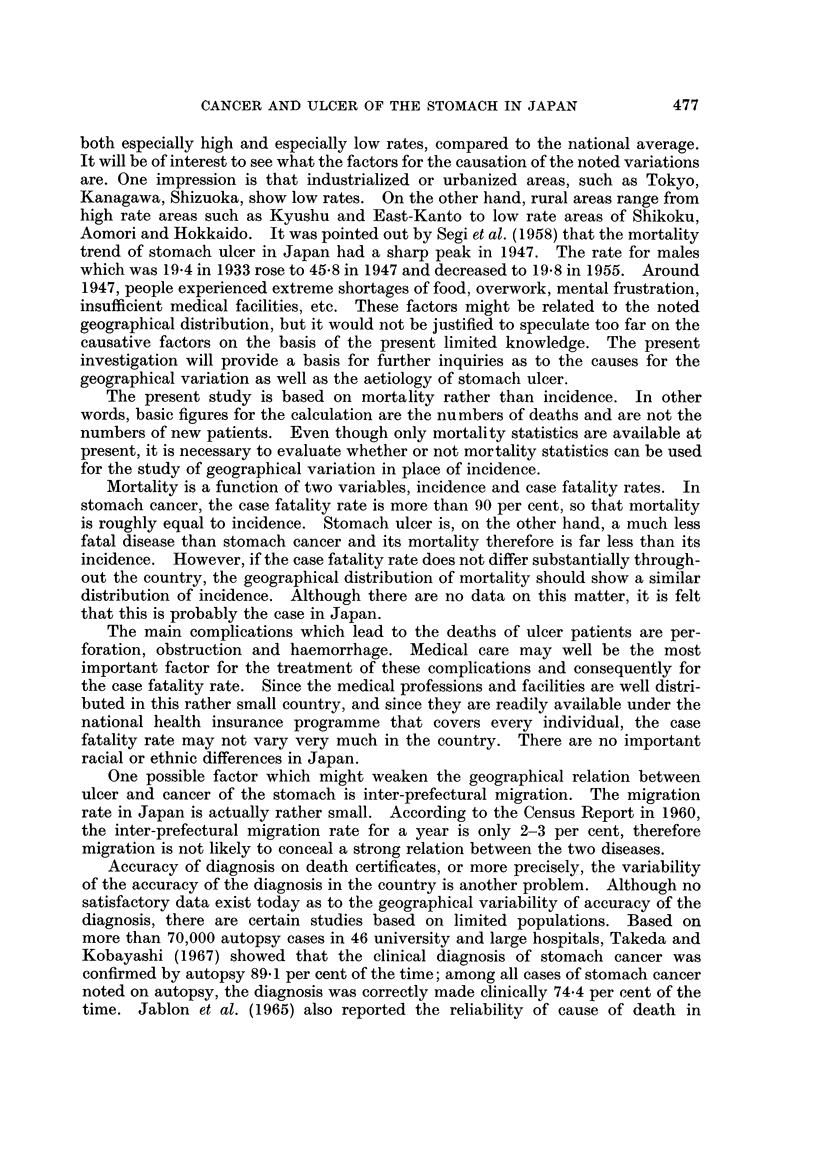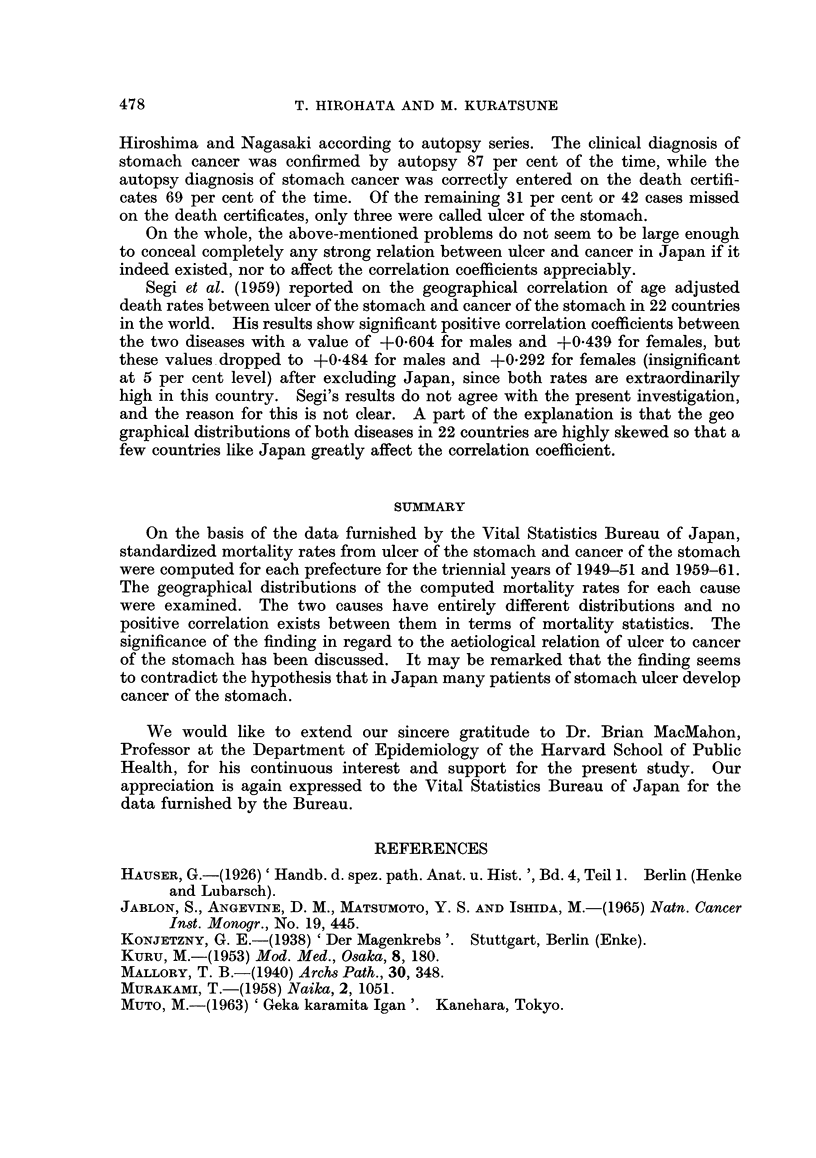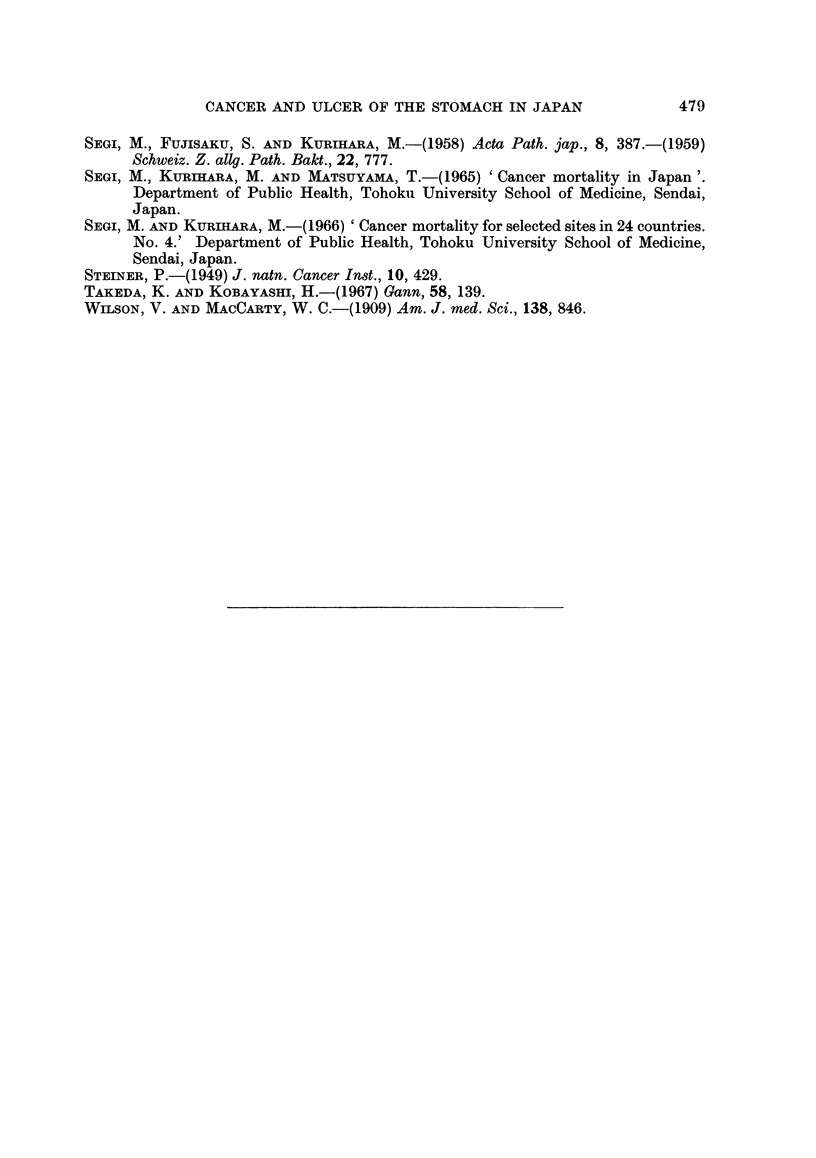# The geographical comparison of mortality from cancer of the stomach and ulcer of the stomach in Japan.

**DOI:** 10.1038/bjc.1969.59

**Published:** 1969-09

**Authors:** T. Hirohata, M. Kuratsune


### BRITISH JOURNAL OF CANCER

VOL. XXIII SEPTEMBER, 1969 NO. 3

### THE GEOGRAPHICAL COMPARISON OF MORTALITY FROM CANCER OF THE STOMACH AND ULCER OF THE STOMACH IN JAPAN

T. HIROHATA AND M. KURATSUNE

*From the Department of Public Health, Faculty of Medicine, Kyushu University, Fukuoka City, Japan*

Received for publication April 9, 1969

It is well known that both cancer of the stomach and ulcer of the stomach are remarkably prevalent in Japan in comparison with other countries. There is well substantiated evidence concerning the high mortality rates from cancer of the stomach (Segi *et al.*, 1965; Segi and Kurihara, 1966; Steiner, 1949). The study of age adjusted mortality rates from cancer of the stomach in 24 countries shows that in 1962–63 Japanese males had the highest rate, 67·96 per 100,000 male population, and Japanese females had the second highest rate, 35·99 per 100,000 female population (Segi and Kurihara, 1966). These rates are approximately seven times as high as those of the white population of the United States for both males and females, who have the lowest rates in the 24 countries.

The mortality rate from ulcer of the stomach in Japan is also extraordinarily high in comparison with other countries. The age adjusted mortality rates per 100,000 population are 27·17 for Japanese males and 9·72 for Japanese females in 1954–55 (Segi *et al.*, 1959). These rates are over three times as high as those of the second highest countries, namely 8·15 for males in Chile and 2·46 for females in Scotland. For ulcer of the duodenum, the national ranking of Japan is very different from ulcer of the stomach. The age adjusted mortality rates from ulcer of the duodenum per 100,000 population in 1954–55 are 2·24 for Japanese males and 0·84 for Japanese females, being ranked as 17th for males and 9th for females among the selected 23 countries (Segi *et al.*, 1959).

The question as to whether any aetiological relationship exists between the two diseases in Japan deserves investigation. The causative relationship of ulcer of the stomach to cancer of the stomach has been a subject of controversy in the world for almost a century (Wilson and MacCarty, 1909; Hauser, 1926; Konjetzny, 1938; Mallory, 1940). The estimated proportion of "ulcer-cancer", *i.e.* cancer which has developed from ulcer, varies considerably from one author to another. It has been noted that the reported rates range from 0 per cent to 100 per cent (Konjetzny, 1938). The problem of ulcer-cancer has also been investigated vigorously in Japan for many years, probably due to the fact that both diseases are strikingly prevalent in the country. Today the problem seems to be one of the most important issues facing pathologists and cancer research workers in Japan.

The rate of ulcer-cancer has been estimated by various authors on the basis of pathological examinations, *e.g.* it was reported as 39·1 per cent among 301 gastric cancer cases (Kuru, 1953), as 9·4 per cent among 1579 gastric cancers and as 24·9 per cent among 600 gastric ulcers (Muto, 1963). Murakami (1958), on the basis of his observed rate of ulcer-cancer of 24·5 per cent, estimated that patients with ulcer of the stomach have a fivefold greater risk of developing cancer of the stomach than do the general populations.

In view of the prevailing strong belief in the existence of the causative relation between ulcer and cancer of the stomach in Japan, it was felt to be significant to investigate the problem from an epidemiologic standpoint. One possible epidemiological way to investigate the relation of the two diseases is to compare the geographical distributions of the incidence rates. The geographical variation of the incidence of ulcer and cancer of the stomach should follow a similar pattern if either of the following hypotheses is true:

(1) Cancer of the stomach in fact develops in many instances from ulcer of the stomach.

(2) The same or similar causative factors play a great role in the causation of the two ailments.

As for cancer of the stomach, its geographical variation as noted in standardized mortality ratios has been investigated by Segi (1965). On the other hand, no studies have been done regarding the geographical distribution of ulcer of the stomach.

Japan consists of 46 administrative regions which are called " Ken " or prefectures. The Vital Statistics Report of Japan does not list the number of deaths from ulcer of the stomach with the detail necessary to compute standardized mortality rates by prefecture. At our request, the Bureau of Vital Statistics, Ministry of Health and Welfare in Japan kindly furnished the list of deaths from ulcer of the stomach by sex and by prefecture for the years of 1949–51 and 1959–61. This made it possible to standardize the mortality rates from ulcer of the stomach by prefecture and thus to determine its geographical distribution and its correlation with the distribution of cancer of the stomach.

### METHODS

According to the Mortality Statistics based on the Vital Statistics Report in Japan and the data that were furnished by the Bureau of Vital Statistics, Ministry of Health and Welfare in Japan, standardized mortality ratios (SMRs) and standardized mortality rates were calculated for ulcer of the stomach and cancer of the stomach for each sex and each prefecture in the triennial periods of 1949–51 and 1959–61 (Tables I and II). The calculation for cancer of the stomach was carried out in spite of the pre-existing report of Segi (1965), because uniformity regarding the method of standardization and the study period involved was needed for comparison between the two diseases.

Since the age breakdown for the number of deaths was not given for each prefecture, age adjustment was conducted by the indirect method. The first step was the selection of a series of standard mortality rates by 5-year-age groups for each cause of death. This was calculated for each cause and for the period of 1949–51 in the whole country, dividing the number of deaths by 5-year-age groups for that period by the 1950 census population of the corresponding age group.

The series of the specific rates was then applied to the census population of each prefecture in 1950 and in 1960 respectively, thus resulting in the expected number of deaths. Annual standardized mortality rates and standardized mortality ratios (SMRs) were then calculated in the usual way, with the results shown in Tables I and II.

FIG. 1.—Map of Japan by prefecture and district.

Geographical correlations were investigated according to different combinations of geographical distributions. It was a matter of course to study the correlation between ulcer and cancer of the stomach, but the correlations between sexes, and between 1949–51 and 1959–61, were also investigated to estimate the constancy of the rates by prefecture and in order to see the geographical correlation in terms of sex and time.

### RESULTS

### *Ulcer of the stomach*

Standardized mortality rates and SMRs for ulcer of the stomach are shown in Table I for each prefecture and sex for the years 1949–51 and 1959–61. The SMRs are illustrated in Fig. 2 and 3. The maps are drawn so that the higher the density of shading, the higher the SMR.

Among males in 1949–51, Kagoshima prefecture had the highest mortality rate, 40·7 per 100,000 population, followed by Tochigi (38·7), Yamaguchi (37·6), Oita

TABLE IA.—*Standardized Mortality Rates and Ratios of Ulcer of the Stomach for the Years of* 1949–51

| | | Males | | | Females | | |
|---|---|---|---|---|---|---|---|
| No. | Prefecture | No. of deaths | Rates* | Ratios | No. of deaths | Rates* | Ratios |
| | All Japan | 37519 | 30·64 | 100·0 | 15011 | 11·80 | 100·0 |
| 1 | Hokkaido | 1168 | 21·05 | 68·7 | 490 | 9·65 | 81·7 |
| 2 | Aomori | 402 | 25·09 | 81·9 | 171 | 10·57 | 89·6 |
| 3 | Iwate | 563 | 30·49 | 99·5 | 255 | 13·67 | 115·8 |
| 4 | Miyagi | 579 | 26·46 | 86·4 | 229 | 10·23 | 86·7 |
| 5 | Akita | 456 | 27·93 | 91·1 | 178 | 10·89 | 92·3 |
| 6 | Yamagata | 637 | 34·32 | 112·0 | 263 | 13·27 | 112·4 |
| 7 | Fukushima | 929 | 32·52 | 106·1 | 365 | 12·17 | 103·1 |
| 8 | Ibaraki | 1156 | 37·26 | 121·6 | 429 | 12·87 | 109·0 |
| 9 | Tochigi | 855 | 38·67 | 126·2 | 288 | 12·11 | 102·6 |
| 10 | Gumma | 774 | 33·39 | 109·0 | 321 | 13·36 | 113·2 |
| 11 | Saitama | 1011 | 31·86 | 104·0 | 422 | 12·85 | 108·9 |
| 12 | Chiba | 1135 | 34·12 | 111·3 | 504 | 13·77 | 116·7 |
| 13 | Tokyo | 2039 | 24·48 | 79·9 | 936 | 11·91 | 100·9 |
| 14 | Kanagawa | 950 | 27·95 | 91·2 | 425 | 12·67 | 107·3 |
| 15 | Niigata | 1056 | 29·83 | 97·4 | 401 | 10·25 | 86·8 |
| 16 | Toyama | 442 | 29·90 | 97·6 | 199 | 12·29 | 104·1 |
| 17 | Ishikawa | 464 | 31·13 | 101·6 | 238 | 14·10 | 119·5 |
| 18 | Fukui | 335 | 27·32 | 89·2 | 122 | 9·19 | 77·8 |
| 19 | Yamanashi | 393 | 32·31 | 105·4 | 179 | 13·74 | 116·4 |
| 20 | Nagano | 977 | 29·79 | 97·2 | 467 | 13·46 | 114·0 |
| 21 | Gifu | 647 | 25·74 | 84·0 | 293 | 11·89 | 100·7 |
| 22 | Shizuoka | 1074 | 29·41 | 96·0 | 395 | 10·59 | 89·7 |
| 23 | Aichi | 1324 | 26·56 | 86·7 | 506 | 9·91 | 83·9 |
| 24 | Mie | 769 | 32·28 | 105·3 | 289 | 11·48 | 97·3 |
| 25 | Shiga | 507 | 35·74 | 116·6 | 156 | 9·96 | 84·4 |
| 26 | Kyoto | 861 | 30·62 | 99·9 | 354 | 11·71 | 99·2 |
| 27 | Osaka | 1677 | 31·46 | 102·7 | 596 | 11·10 | 94·0 |
| 28 | Hyogo | 1482 | 29·58 | 96·5 | 549 | 10·68 | 90·5 |
| 29 | Nara | 450 | 36·56 | 119·3 | 122 | 9·27 | 78·5 |
| 30 | Wakayama | 513 | 31·54 | 102·9 | 164 | 9·43 | 79·9 |
| 31 | Tottori | 335 | 34·33 | 112·0 | 105 | 9·59 | 81·2 |
| 32 | Shimane | 578 | 35·99 | 117·4 | 224 | 12·98 | 110·0 |
| 33 | Okayama | 842 | 29·42 | 96·0 | 349 | 11·74 | 99·5 |
| 34 | Hiroshima | 1112 | 32·81 | 107·1 | 448 | 12·47 | 105·6 |
| 35 | Yamaguchi | 931 | 37·58 | 122·6 | 365 | 14·37 | 121·7 |
| 36 | Tokushima | 397 | 27·27 | 89·0 | 175 | 11·35 | 96·2 |
| 37 | Kagawa | 497 | 32·21 | 105·1 | 176 | 10·82 | 91·7 |
| 38 | Ehime | 610 | 25·38 | 82·8 | 240 | 9·49 | 80·4 |
| 39 | Kochi | 362 | 23·22 | 75·8 | 149 | 9·01 | 76·3 |
| 40 | Fukuoka | 1570 | 32·41 | 105·8 | 578 | 11·48 | 97·3 |
| 41 | Saga | 466 | 33·71 | 110·0 | 199 | 13·17 | 111·5 |
| 42 | Nagasaki | 755 | 32·13 | 104·8 | 292 | 11·50 | 97·4 |
| 43 | Kumamoto | 1014 | 36·27 | 118.4 | 442 | 14·38 | 121·8 |
| 44 | Oita | 752 | 37·31 | 121·8 | 349 | 16·46 | 139·4 |
| 45 | Miyazaki | 588 | 37·31 | 121·7 | 200 | 12·51 | 106·0 |
| 46 | Kagoshima | 1057 | 40·67 | 132·7 | 413 | 13·81 | 117·0 |
| | Unknown | 28 | — | — | 1 | — | — |

* Annual average rates per 100,000 population.

TABLE IB.—*Standardized Mortality Rates and Ratios of Ulcer of the Stomach for the Years of* 1959–61

| No. | Prefecture | Males No. of deaths | Males Rates* | Males Ratios | Females No. of deaths | Females Rates* | Females Ratios |
|---|---|---|---|---|---|---|---|
| | All Japan | 20474 | 13·25 | 100·0 | 9402 | 5·79 | 100·0 |
| 1 | Hokkaido | 724 | 9·91 | 74·8 | 307 | 4·50 | 77·8 |
| 2 | Aomori | 233 | 11·61 | 87·6 | 80 | 3·80 | 65·7 |
| 3 | Iwate | 282 | 12·54 | 94·6 | 159 | 6·82 | 117·8 |
| 4 | Miyagi | 308 | 11·35 | 85·7 | 153 | 5·34 | 92·2 |
| 5 | Akita | 221 | 10·96 | 82·7 | 102 | 4·87 | 84·1 |
| 6 | Yamagata | 306 | 13·86 | 104·6 | 139 | 5·76 | 99·5 |
| 7 | Fukushima | 466 | 13·97 | 105·4 | 204 | 5·68 | 98·0 |
| 8 | Ibaraki | 663 | 18·61 | 140·4 | 279 | 7·24 | 125·1 |
| 9 | Tochigi | 488 | 19·00 | 143·4 | 216 | 7·75 | 133·9 |
| 10 | Gumma | 409 | 14·93 | 112·7 | 199 | 6·88 | 118·9 |
| 11 | Saitama | 556 | 14·16 | 106·8 | 278 | 6·84 | 118·1 |
| 12 | Chiba | 665 | 16·60 | 125·2 | 323 | 7·43 | 128·3 |
| 13 | Tokyo | 1302 | 9·53 | 71·9 | 635 | 4·76 | 82·2 |
| 14 | Kanagawa | 511 | 9·98 | 75·3 | 236 | 4·63 | 80·0 |
| 15 | Niigata | 535 | 13·01 | 98·1 | 224 | 4·90 | 84·6 |
| 16 | Toyama | 180 | 10·09 | 76·1 | 98 | 5·06 | 87·4 |
| 17 | Ishikawa | 199 | 11·57 | 87·3 | 118 | 6·09 | 105·1 |
| 18 | Fukui | 180 | 12·91 | 97·4 | 102 | 6·66 | 115·1 |
| 19 | Yamanashi | 201 | 14·16 | 106·9 | 114 | 7·43 | 128·2 |
| 20 | Nagano | 508 | 13·36 | 100·8 | 249 | 6·11 | 105·6 |
| 21 | Gifu | 319 | 10·73 | 80·9 | 183 | 6·12 | 105·7 |
| 22 | Shizuoka | 612 | 13·43 | 101·3 | 281 | 5·92 | 102·2 |
| 23 | Aichi | 694 | 10·54 | 79·5 | 317 | 4·69 | 81·1 |
| 24 | Mie | 372 | 13·30 | 100·3 | 158 | 5·28 | 91·1 |
| 25 | Shiga | 263 | 16·31 | 123·0 | 124 | 6·92 | 119·5 |
| 26 | Kyoto | 487 | 13·62 | 102·8 | 214 | 5·52 | 95·4 |
| 27 | Osaka | 1035 | 12·62 | 95·2 | 422 | 5·05 | 87·2 |
| 28 | Hyogo | 914 | 13·79 | 104·0 | 394 | 5·69 | 98·3 |
| 29 | Nara | 246 | 16·99 | 128·2 | 89 | 5·75 | 99·3 |
| 30 | Wakayama | 230 | 11·89 | 89·7 | 109 | 5·24 | 90·5 |
| 31 | Tottori | 155 | 13·72 | 103·5 | 54 | 4·20 | 72·6 |
| 32 | Shimane | 326 | 18·16 | 137·0 | 165 | 8·48 | 146·5 |
| 33 | Okayama | 455 | 13·79 | 104·0 | 183 | 5·24 | 90·5 |
| 34 | Hiroshima | 503 | 12·41 | 93·6 | 238 | 5·46 | 94·4 |
| 35 | Yamaguchi | 469 | 16·03 | 120·9 | 223 | 7·28 | 125·7 |
| 36 | Tokushima | 210 | 13·03 | 98·3 | 95 | 5·51 | 95·2 |
| 37 | Kagawa | 220 | 12·59 | 95·0 | 90 | 4·74 | 81·8 |
| 38 | Ehime | 336 | 12·26 | 92·5 | 149 | 5·04 | 87·0 |
| 39 | Kochi | 191 | 10·77 | 81·2 | 94 | 4·88 | 84·3 |
| 40 | Fukuoka | 886 | 14·19 | 107·1 | 426 | 6·42 | 110·8 |
| 41 | Saga | 204 | 12·79 | 96·5 | 86 | 4·84 | 83·6 |
| 42 | Nagasaki | 505 | 18·07 | 136·3 | 205 | 6·74 | 116·4 |
| 43 | Kumamoto | 512 | 15·77 | 119·0 | 283 | 7·88 | 136·2 |
| 44 | Oita | 386 | 17·00 | 128·2 | 182 | 7·46 | 128·9 |
| 45 | Miyazaki | 292 | 15·57 | 117·5 | 141 | 7·20 | 124·3 |
| 46 | Kagoshima | 664 | 19·50 | 147·1 | 276 | 6·88 | 118·8 |
| | Unknown | 51 | — | — | 6 | — | — |

* Annual average rates per 100,000 population.

TABLE IIA.—*Standardized Mortality Rates and Ratios of Cancer of the Stomach for the Years of* 1949–51

| | | Males | | | Females | | |
|---|---|---|---|---|---|---|---|
| No. | Prefecture | No. of deaths | Rates* | Ratios | No. of deaths | Rates* | Ratios |
| | All Japan | 56669 | 46·28 | 100·0 | 35766 | 28·13 | 100·0 |
| 1 | Hokkaido | 2518 | 46·69 | 100·9 | 1359 | 27·33 | 97·2 |
| 2 | Aomori | 638 | 40·72 | 88·0 | 433 | 26·80 | 95·3 |
| 3 | Iwate | 525 | 28·52 | 61·6 | 381 | 20·40 | 72·5 |
| 4 | Miyagi | 1112 | 51·40 | 111·1 | 651 | 29·10 | 103·5 |
| 5 | Akita | 795 | 50·06 | 108·2 | 535 | 32·50 | 115·6 |
| 6 | Yamagata | 1166 | 63·14 | 136·4 | 736 | 36·59 | 130·1 |
| 7 | Fukushima | 1302 | 45·41 | 98·1 | 878 | 29·13 | 103·6 |
| 8 | Ibaraki | 1503 | 47·70 | 103·1 | 982 | 29·47 | 104·8 |
| 9 | Tochigi | 1140 | 51·16 | 110·5 | 777 | 32·50 | 115·6 |
| 10 | Gumma | 1267 | 54·33 | 117·4 | 820 | 33·80 | 120·2 |
| 11 | Saitama | 1671 | 52·33 | 113·1 | 1101 | 33·32 | 118·5 |
| 12 | Chiba | 1802 | 53·42 | 115·4 | 1154 | 31·49 | 112·0 |
| 13 | Tokyo | 3946 | 50·05 | 108·1 | 2393 | 30·55 | 108·6 |
| 14 | Kanagawa | 1634 | 49·75 | 107·5 | 994 | 29·68 | 105·5 |
| 15 | Niigata | 2161 | 60·45 | 130·6 | 1496 | 37·79 | 134·4 |
| 16 | Toyama | 890 | 60·02 | 129·7 | 568 | 34·84 | 123·9 |
| 17 | Ishikawa | 812 | 53·58 | 115·8 | 514 | 30·18 | 107·3 |
| 18 | Fukui | 520 | 41·40 | 89·5 | 368 | 27·56 | 98·0 |
| 19 | Yamanashi | 546 | 44·12 | 95·3 | 333 | 25·40 | 90·3 |
| 20 | Nagano | 1765 | 52·65 | 113·8 | 1089 | 30·88 | 109·8 |
| 21 | Gifu | 1007 | 39·14 | 84·6 | 729 | 29·52 | 105·0 |
| 22 | Shizuoka | 1258 | 34·15 | 73·8 | 851 | 22·83 | 81·2 |
| 23 | Aichi | 2319 | 46·40 | 100·3 | 1552 | 30·42 | 108·2 |
| 24 | Mie | 1128 | 46·15 | 99·7 | 712 | 28·07 | 99·8 |
| 25 | Shiga | 638 | 43·77 | 94·6 | 449 | 28·35 | 100·8 |
| 26 | Kyoto | 1360 | 48·44 | 104·7 | 863 | 28·26 | 100·5 |
| 27 | Osaka | 2869 | 55·88 | 120·7 | 1631 | 30·27 | 107·6 |
| 28 | Hyogo | 2286 | 45·91 | 99·2 | 1339 | 26·02 | 92·5 |
| 29 | Nara | 864 | 69·11 | 149·3 | 533 | 40·04 | 142·4 |
| 30 | Wakayama | 787 | 47·40 | 102·4 | 488 | 27·96 | 99·4 |
| 31 | Tottori | 575 | 57·49 | 124·2 | 341 | 31·22 | 111·0 |
| 32 | Shimane | 627 | 37·57 | 81·2 | 397 | 23·07 | 82·0 |
| 33 | Okayama | 1227 | 41·71 | 90·1 | 771 | 25·92 | 92·1 |
| 34 | Hiroshima | 1374 | 39·87 | 86·2 | 883 | 24·75 | 88·0 |
| 35 | Yamaguchi | 1018 | 40·53 | 87·6 | 631 | 24·88 | 88·4 |
| 36 | Tokushima | 549 | 36·67 | 79·2 | 318 | 20·92 | 74·4 |
| 37 | Kagawa | 678 | 42·76 | 92·4 | 443 | 27·31 | 97·1 |
| 38 | Ehime | 1050 | 42·86 | 92·6 | 628 | 25·15 | 89·4 |
| 39 | Kochi | 597 | 37·25 | 80·5 | 331 | 20·10 | 71·5 |
| 40 | Fukuoka | 2398 | 50·62 | 109·4 | 1450 | 29·00 | 103·1 |
| 41 | Saga | 699 | 50·17 | 108·4 | 462 | 30·40 | 108·1 |
| 42 | Nagasaki | 864 | 37·00 | 80·0 | 539 | 21·48 | 76·4 |
| 43 | Kumamoto | 878 | 30·89 | 66·7 | 661 | 21·63 | 76·9 |
| 44 | Oita | 713 | 34·43 | 74·4 | 442 | 20·84 | 74·1 |
| 45 | Miyazaki | 539 | 33·98 | 73·4 | 309 | 19·57 | 69·6 |
| 46 | Kagoshima | 637 | 24·23 | 52·4 | 450 | 15·12 | 53·7 |
| | Unknown | 17 | — | — | 1 | — | — |

* Annual average rates per 100,000 population.

TABLE IIB.—*Standardized Mortality Rates and Ratios of Cancer of the Stomach for the Years* 1959–61

| No. | Prefecture | Males: No. of deaths | Males: Rates* | Males: Ratios | Females: No. of deaths | Females: Rates* | Females: Ratios |
|---|---|---|---|---|---|---|---|
| | All Japan | 78509 | 50·40 | 100·0 | 49366 | 30·62 | 100·0 |
| 1 | Hokkaido | 3470 | 48·66 | 96·6 | 1875 | 28·10 | 91·8 |
| 2 | Aomori | 916 | 45·96 | 91·2 | 617 | 29·42 | 96·1 |
| 3 | Iwate | 755 | 33·35 | 66·2 | 512 | 22·07 | 72·1 |
| 4 | Miyagi | 1391 | 50·88 | 101·0 | 831 | 29·16 | 95·2 |
| 5 | Akita | 1282 | 63·37 | 125·7 | 686 | 32·24 | 105·3 |
| 6 | Yamagata | 1484 | 65·86 | 130·7 | 891 | 36·38 | 118·8 |
| 7 | Fukushima | 1847 | 54·22 | 107·6 | 1131 | 31·40 | 102·6 |
| 8 | Ibaraki | 1845 | 50·53 | 100·3 | 1145 | 29·90 | 97·7 |
| 9 | Tochigi | 1560 | 59·36 | 117·8 | 977 | 35·11 | 114·7 |
| 10 | Gumma | 1564 | 55·71 | 110·5 | 1000 | 34·51 | 112·7 |
| 11 | Saitama | 2344 | 59·34 | 117·7 | 1456 | 35·99 | 117·5 |
| 12 | Chiba | 2301 | 56·38 | 111·9 | 1460 | 33·81 | 110·4 |
| 13 | Tokyo | 6875 | 52·73 | 104·6 | 4303 | 32·67 | 106·7 |
| 14 | Kanagawa | 2524 | 50·90 | 101·0 | 1590 | 31·56 | 103·1 |
| 15 | Niigata | 2603 | 62·22 | 123·5 | 1895 | 41·40 | 135·2 |
| 16 | Toyama | 1078 | 59·70 | 118·5 | 773 | 39·98 | 130·6 |
| 17 | Ishikawa | 990 | 56·30 | 111·7 | 668 | 34·59 | 113·0 |
| 18 | Fukui | 621 | 43·15 | 85·6 | 413 | 27·20 | 88·8 |
| 19 | Yamanashi | 734 | 50·39 | 100·0 | 451 | 29·50 | 96·3 |
| 20 | Nagano | 2100 | 53·37 | 105·9 | 1273 | 31·02 | 101·3 |
| 21 | Gifu | 1470 | 48·25 | 95·7 | 935 | 31·67 | 103·4 |
| 22 | Shizuoka | 1774 | 38·50 | 76·4 | 1148 | 24·40 | 79·7 |
| 23 | Aichi | 3296 | 50·26 | 99·7 | 2211 | 33·32 | 108·8 |
| 24 | Mie | 1439 | 49·82 | 98·9 | 875 | 29·34 | 95·8 |
| 25 | Shiga | 878 | 52·40 | 104·0 | 559 | 31·30 | 102·2 |
| 26 | Kyoto | 1908 | 52·22 | 103·6 | 1193 | 30·79 | 100·6 |
| 27 | Osaka | 4628 | 57·74 | 114·6 | 2652 | 31·86 | 104·1 |
| 28 | Hyogo | 3312 | 49·63 | 98·5 | 2058 | 29·90 | 97·7 |
| 29 | Nara | 1021 | 68·79 | 136·5 | 592 | 38·16 | 124·6 |
| 30 | Wakayama | 1102 | 55·20 | 109·5 | 640 | 30·98 | 101·2 |
| 31 | Tottori | 607 | 51·90 | 103·0 | 437 | 34·35 | 112·2 |
| 32 | Shimane | 839 | 44·70 | 88·7 | 578 | 30·15 | 98·5 |
| 33 | Okayama | 1497 | 43·62 | 86·6 | 885 | 25·54 | 83·4 |
| 34 | Hiroshima | 1946 | 46·88 | 93·0 | 1258 | 29·25 | 95·5 |
| 35 | Yamaguchi | 1429 | 47·59 | 94·4 | 932 | 30·61 | 100·0 |
| 36 | Tokushima | 656 | 39·28 | 77·9 | 461 | 27·22 | 88·9 |
| 37 | Kagawa | 933 | 51·62 | 102·4 | 601 | 31·92 | 104·3 |
| 38 | Ehime | 1427 | 50·42 | 100·0 | 849 | 29·25 | 95·5 |
| 39 | Kochi | 772 | 41·47 | 82·3 | 461 | 23·91 | 78·1 |
| 40 | Fukuoka | 3199 | 51·30 | 101·8 | 2037 | 30·95 | 101·1 |
| 41 | Saga | 932 | 56·68 | 112·5 | 606 | 34·07 | 111·3 |
| 42 | Nagasaki | 1134 | 40·00 | 79·4 | 759 | 25·17 | 82·2 |
| 43 | Kumamoto | 1209 | 35·99 | 71·4 | 852 | 23·94 | 78·2 |
| 44 | Oita | 929 | 39·39 | 78·2 | 605 | 24·96 | 81·5 |
| 45 | Miyazaki | 781 | 40·80 | 81·0 | 494 | 25·39 | 82·9 |
| 46 | Kagoshima | 990 | 28·06 | 55·7 | 720 | 18·07 | 59·0 |
| | Unknown | 117 | — | — | 21 | — | — |

* Annual average rates per 100,000 population.

(37·3), Miyazaki (37·3), and Ibaraki (37·3). On the other hand, Hokkaido had the lowest rate (21·1), followed by Kochi (23·2), and Tokyo (24·5).

On the whole, high rate areas were clustered in the Kyushu District, which is the southern-most island, and in the East-Kanto District, which is located in the central part of Japan facing the Pacific Ocean; Hokkaido, Shikoku District and Tokyo were low areas. The average annual rate for the whole country was 30·6 per 100,000 population.

For females, in the same years of 1949–51, Oita had the highest rate of 16·5 per 100,000, followed by Kumamoto (14·4) and Yamaguchi (14·4). Kochi had the lowest rate (9·0), followed by Fukui (9·2), Nara (9·3), and Wakayama (9·4). The annual average rate for the whole country was 11·8 per 100,000 female population. As a whole, Kyushu District again showed high rates while West-Shikoku District, Nara, and Wakayama prefectures had low rates.

The geographical distribution did not coincide exactly for the two sexes, but a similar pattern of the distribution is evident. The ratio for the country of male to female rates is 2·6 to 1, indicating that ulcer of the stomach prevails far more in males than in females.

As was the case in 1949–51, Kagoshima prefecture had the highest mortality rates for males in 1959–61, *i.e.* 19·5 per 100,000, followed by Tochigi (19·0) and Ibaraki (18·6). These rates were more than 40 per cent higher than the average of the country. Shimane (18·2) and Nagasaki (18·1) came next with rates of more than 30 per cent higher and Oita (17·0), Nara (17·0), Chiba (16·6), Shiga (16·3) and Yamaguchi (16·0) had rates at least 20 per cent above the national average. Tokyo had the lowest rate of 9·5 per 100,000 population followed by Hokkaido (9·9), Kanagawa (10·0), Toyama (10·1) and Aichi (10·5). These prefectures had rates at least 20 per cent lower than the national average, which was 13·3.

It will be noted on the map that, in general, East-Kanto District and Kyushu District with adjacent Yamaguchi and Shimane prefectures had high rates while Tokyo and Kanagawa, Hokkaido, Chubu had low rates. In comparison of the distribution in 1949–51 with that in 1959–61, the distribution, in general, seems to remain unchanged, although a remarkable decrease in the rates took place over this period, namely, a 56·7 per cent decrease in the national average.

Among females in 1959–61, Shimane had the highest rate of 8·5 per 100,000, more than 40 per cent higher than the national average of 5·8, followed by Kumamoto (7·9) and Tochigi (7·8). Oita (7·5), Chiba (7·4), Yamanashi (7·4), Yamaguchi (7·3), Ibaraki (7·2) and Miyazaki (7·2) had rates more than 20 per cent higher than the national average.

The lowest rate is 3·8 in Aomori, followed by Tottori (4·2), and Hokkaido (4·5), which were more than 20 per cent lower than the country average. On the whole, East-Kanto, Kyushu with adjacent Yamaguchi and Shimane tended to be high while Hokkaido and Aomori showed low rates.

The distribution of female rates in 1959–61 indicates a similar pattern to that of the period of 1949–51, but was more closely related to that of males in 1959–61. A remarkable decrease was also noted for females, namely, the average rate of the country in 1959–61 was only 49·0 per cent of that in 1949–51. The male to female ratio was 2·29, indicating a male predominance of the disease.

The common pattern of the distribution, seen in both sexes and in the two periods studied, is that East-Kanto District (Tochigi, Ibaraki and Chiba pre-

**Fig. 2.—Standardized mortality ratios from ulcer of the stomach by sex and prefecture in Japan, 1949–51.**

**Fig. 3.—Standardized mortality ratios from ulcer of the stomach by sex and prefecture in Japan, 1959–61.**

fectures) and Kyushu District have high rates, while Hokkaido, Aomori, Tokyo, Aichi prefectures and Shikoku District show low rates.

*Cancer of the stomach*

As described before, Segi (1965) investigated the geographical distribution of the mortality from cancer of the stomach in Japan for the years of 1950–59. The results obtained by the present study, shown in Table II and Fig. 4 and 5, are quite similar to those of Segi although the standard population and the period of years studied are different, so that the results will be described here briefly.

Regardless of sex and the two time periods, almost the same pattern of geographical distribution will be seen in Fig. 4 and 5. Prefectures with high rates of cancer of the stomach are concentrated on the north-eastern part of Honshu along the coast of the Sea of Japan, including Yamagata, Niigata and Toyama, etc. Apart from the above prefectures, Nara, a prefecture of the Kinki District, had a high rate of cancer of the stomach.

Low-rate prefectures are concentrated in Kyushu District (Kagoshima, Kumamoto, Miyazaki, Oita and Nagasaki prefectures) and in Shikoku District (Tokushima and Kochi prefectures). Iwate (Tohoku District) and Shizuoka (Chubu District) prefectures are also favoured ones. It will be noticed in the four maps for the different sexes and different years that the geographical distribution is surprisingly constant for all.

*Geographical correlation*

Geographical correlations between the various combinations of diseases, sexes and different periods of years are presented as correlation coefficients in Table III.

There appears to be no geographical correlation between the mortality from ulcer of the stomach and that from cancer of the stomach as seen in Table IIIA

TABLE III.—*Correlation Coefficients Between Mortality Rates of Selected Combinations*

A. Stomach ulcer and stomach cancer, male

| | | Stomach ulcer | |
|---|---|---|---|
| | | 1949–51 | 1959–61 |
| Stomach cancer | 1949–51 | −0·051 | — |
| | 1959–61 | −0·017 | −0·187 |

B. Stomach ulcer and stomach cancer, female

| | | Stomach ulcer | |
|---|---|---|---|
| | | 1949–51 | 1959–61 |
| Stomach cancer | 1949–51 | −0·198 | — |
| | 1959–61 | −0·134 | −0·247 |

C. Stomach ulcer, 1949–51 *vs.* 1959–61

| | | 1949–51 | |
|---|---|---|---|
| | | Male | Female |
| 1959–61 | Male | +0·844 | — |
| | Female | — | +0·595 |

D. Stomach cancer, 1949–51 *vs.* 1959–61

| | | 1949–51 | |
|---|---|---|---|
| | | Male | Female |
| 1959–61 | Male | +0·932 | — |
| | Female | — | +0·925 |

E. Stomach ulcer, male *vs.* female

| | | Male | |
|---|---|---|---|
| | | 1949–51 | 1959–61 |
| Female | 1949–51 | +0·532 | — |
| | 1959–61 | — | +0·762 |

F. Stomach cancer, male *vs.* female

| | | Male | |
|---|---|---|---|
| | | 1949–51 | 1959–61 |
| Female | 1949–51 | +0·942 | — |
| | 1959–61 | — | +0·917 |

**FIG. 4.—Standardized mortality ratios from cancer of the stomach by sex and prefecture in Japan, 1949–51.**

**FIG. 5.—Standardized mortality ratios from cancer of the stomach by sex and prefecture in Japan, 1959–61.**

and B. Correlation coefficients between the two diseases were calculated based on the distribution of the same sex and the same period of years resulting in four figures according to the four combinations. Also, correlation between ulcer of the stomach in 1949–51 and cancer of the stomach in 1959–61 was examined because a substantial number of years would be needed for the development from ulcer to cancer if such relation should exist. All of the six figures are close to zero, leading to the conclusion that the null hypothesis, namely, no geographical correlation between the mortality from ulcer of the stomach and cancer of the stomach cannot be rejected. Thus, this result does not support the claim that the two ailments have strong causative association. It is of interest to note that all six figures tend to have negative values.

Table IIIC and D express the geographical correlation of mortality in terms of the passage of ten years. As to stomach cancer, the correlation coefficient is + 0·932 for males and +0·925 for females, indicating very little change in the geographical distribution over ten years. As to stomach ulcer, the correlation coefficient is +0·844 for males and +0·595 for females showing high correlation, but not as high as for stomach cancer.

In Table III E and F, correlation is indicated in terms of the relation between the sexes. Again, the distribution of the mortality from stomach cancer is very stable for the different sexes with the correlation coefficient as high as +0·942 for 1949–51 and +0·917 for 1959–61. For stomach ulcer, the correlation coefficient is +0·532 for 1949–51 and +0·762 for 1959–61, showing highly significant values between the distributions of different sexes, but not as high as those for stomach cancer.

*Conclusions*

The conclusions regarding the geographical distributions of mortality from ulcer of the stomach and cancer of the stomach with their geographical associations may be summarized as follows:

1. No significant geographical correlation of mortality was found between ulcer of the stomach and cancer of the stomach. This result does not support the hypothesis of close causative relation between the two diseases.
2. There is a highly positive geographical correlation of cancer of the stomach between males *vs.* females and between 1949–51 *vs.* 1959–61. Ulcer of the stomach also shows high positive correlation between the sexes and between the two periods of years studied, but not as high as that for cancer of the stomach.
3. High rate areas of mortality from ulcer of the stomach are, on the whole, clustered in East-Kanto and Kyushu District, while Hokkaido, Aomori, Tokyo, Aichi prefectures and Shikoku District show low rates.
4. High rate areas of mortality from cancer of the stomach are concentrated on the northeastern part of Honshu along the coast of the Sea of Japan, while low rate areas are concentrated in Kyushu and Shikoku District. Apart from these areas Nara prefecture shows a high rate, while Shizuoka and Iwate prefectures show low rates.

### DISCUSSION

The present investigation reveals that there are substantial geographical variations in Japan in the mortality of ulcer of the stomach, showing areas of both especially high and especially low rates, compared to the national average. It will be of interest to see what the factors for the causation of the noted variations are. One impression is that industrialized or urbanized areas, such as Tokyo, Kanagawa, Shizuoka, show low rates. On the other hand, rural areas range from high rate areas such as Kyushu and East-Kanto to low rate areas of Shikoku, Aomori and Hokkaido. It was pointed out by Segi *et al.* (1958) that the mortality trend of stomach ulcer in Japan had a sharp peak in 1947. The rate for males which was 19·4 in 1933 rose to 45·8 in 1947 and decreased to 19·8 in 1955. Around 1947, people experienced extreme shortages of food, overwork, mental frustration, insufficient medical facilities, etc. These factors might be related to the noted geographical distribution, but it would not be justified to speculate too far on the causative factors on the basis of the present limited knowledge. The present investigation will provide a basis for further inquiries as to the causes for the geographical variation as well as the aetiology of stomach ulcer.

The present study is based on mortality rather than incidence. In other words, basic figures for the calculation are the numbers of deaths and are not the numbers of new patients. Even though only mortality statistics are available at present, it is necessary to evaluate whether or not mortality statistics can be used for the study of geographical variation in place of incidence.

Mortality is a function of two variables, incidence and case fatality rates. In stomach cancer, the case fatality rate is more than 90 per cent, so that mortality is roughly equal to incidence. Stomach ulcer is, on the other hand, a much less fatal disease than stomach cancer and its mortality therefore is far less than its incidence. However, if the case fatality rate does not differ substantially throughout the country, the geographical distribution of mortality should show a similar distribution of incidence. Although there are no data on this matter, it is felt that this is probably the case in Japan.

The main complications which lead to the deaths of ulcer patients are perforation, obstruction and haemorrhage. Medical care may well be the most important factor for the treatment of these complications and consequently for the case fatality rate. Since the medical professions and facilities are well distributed in this rather small country, and since they are readily available under the national health insurance programme that covers every individual, the case fatality rate may not vary very much in the country. There are no important racial or ethnic differences in Japan.

One possible factor which might weaken the geographical relation between ulcer and cancer of the stomach is inter-prefectural migration. The migration rate in Japan is actually rather small. According to the Census Report in 1960, the inter-prefectural migration rate for a year is only 2–3 per cent, therefore migration is not likely to conceal a strong relation between the two diseases.

Accuracy of diagnosis on death certificates, or more precisely, the variability of the accuracy of the diagnosis in the country is another problem. Although no satisfactory data exist today as to the geographical variability of accuracy of the diagnosis, there are certain studies based on limited populations. Based on more than 70,000 autopsy cases in 46 university and large hospitals, Takeda and Kobayashi (1967) showed that the clinical diagnosis of stomach cancer was confirmed by autopsy 89·1 per cent of the time; among all cases of stomach cancer noted on autopsy, the diagnosis was correctly made clinically 74·4 per cent of the time. Jablon *et al.* (1965) also reported the reliability of cause of death in

Hiroshima and Nagasaki according to autopsy series. The clinical diagnosis of stomach cancer was confirmed by autopsy 87 per cent of the time, while the autopsy diagnosis of stomach cancer was correctly entered on the death certificates 69 per cent of the time. Of the remaining 31 per cent or 42 cases missed on the death certificates, only three were called ulcer of the stomach.

On the whole, the above-mentioned problems do not seem to be large enough to conceal completely any strong relation between ulcer and cancer in Japan if it indeed existed, nor to affect the correlation coefficients appreciably.

Segi *et al.* (1959) reported on the geographical correlation of age adjusted death rates between ulcer of the stomach and cancer of the stomach in 22 countries in the world. His results show significant positive correlation coefficients between the two diseases with a value of +0·604 for males and +0·439 for females, but these values dropped to +0·484 for males and +0·292 for females (insignificant at 5 per cent level) after excluding Japan, since both rates are extraordinarily high in this country. Segi's results do not agree with the present investigation, and the reason for this is not clear. A part of the explanation is that the geo graphical distributions of both diseases in 22 countries are highly skewed so that a few countries like Japan greatly affect the correlation coefficient.

### SUMMARY

On the basis of the data furnished by the Vital Statistics Bureau of Japan, standardized mortality rates from ulcer of the stomach and cancer of the stomach were computed for each prefecture for the triennial years of 1949–51 and 1959–61. The geographical distributions of the computed mortality rates for each cause were examined. The two causes have entirely different distributions and no positive correlation exists between them in terms of mortality statistics. The significance of the finding in regard to the aetiological relation of ulcer to cancer of the stomach has been discussed. It may be remarked that the finding seems to contradict the hypothesis that in Japan many patients of stomach ulcer develop cancer of the stomach.

We would like to extend our sincere gratitude to Dr. Brian MacMahon, Professor at the Department of Epidemiology of the Harvard School of Public Health, for his continuous interest and support for the present study. Our appreciation is again expressed to the Vital Statistics Bureau of Japan for the data furnished by the Bureau.

### REFERENCES

HAUSER, G.—(1926) ' Handb. d. spez. path. Anat. u. Hist. ', Bd. 4, Teil 1. Berlin (Henke and Lubarsch).

JABLON, S., ANGEVINE, D. M., MATSUMOTO, Y. S. AND ISHIDA, M.—(1965) *Natn. Cancer Inst. Monogr.*, No. 19, 445.

KONJETZNY, G. E.—(1938) ' Der Magenkrebs '. Stuttgart, Berlin (Enke).

KURU, M.—(1953) *Mod. Med., Osaka*, **8**, 180.

MALLORY, T. B.—(1940) *Archs Path.*, **30**, 348.

MURAKAMI, T.—(1958) *Naika*, **2**, 1051.

MUTO, M.—(1963) ' Geka karamita Igan '. Kanehara, Tokyo.

SEGI, M., FUJISAKU, S. AND KURIHARA, M.—(1958) *Acta Path. jap.*, **8**, 387.—(1959) *Schweiz. Z. allg. Path. Bakt.*, **22**, 777.
SEGI, M., KURIHARA, M. AND MATSUYAMA, T.—(1965) ' Cancer mortality in Japan '. Department of Public Health, Tohoku University School of Medicine, Sendai, Japan.
SEGI, M. AND KURIHARA, M.—(1966) ' Cancer mortality for selected sites in 24 countries. No. 4.' Department of Public Health, Tohoku University School of Medicine, Sendai, Japan.
STEINER, P.—(1949) *J. natn. Cancer Inst.*, **10**, 429.
TAKEDA, K. AND KOBAYASHI, H.—(1967) *Gann*, **58**, 139.
WILSON, V. AND MACCARTY, W. C.—(1909) *Am. J. med. Sci.*, **138**, 846.